# Paired associative stimulation with a high-intensity cortical component and a high-frequency peripheral component in treatment of neuropathic pain after incomplete spinal cord injury – a pilot trial

**DOI:** 10.1038/s41394-026-00729-1

**Published:** 2026-03-04

**Authors:** Kirsi Holopainen, Markus Pohjonen, Erika Kirveskari, Selja Vaalto, Jyrki P. Mäkelä, Jari Arokoski, Anastasia Shulga

**Affiliations:** 1 BioMag Laboratory, HUS Diagnostic Center, Helsinki University Hospital, University of Helsinki and Aalto University School of Science, Helsinki, Finland; 2 Department of Internal Medicine and Rehabilitation, Division of Rehabilitation, Helsinki University Hospital and Helsinki University, Helsinki, Finland; 3 HUS Diagnostic Center, Clinical Neurophysiology, Clinical Neurosciences, Helsinki University Hospital and University of Helsinki, Helsinki, Finland

**Keywords:** Spinal cord diseases, Chronic pain

## Abstract

**Study design:**

Prospective interventional sham-controlled pilot study.

**Objectives:**

To investigate the effect of motor-tract paired-associative stimulation consisting of high-intensity transcranial magnetic stimulation and high-frequency electric stimulation of peripheral nerves (high-PAS) on moderate-to-severe upper limb neuropathic pain in patients with incomplete spinal cord injury compared with sham treatment in the same patients.

**Setting:**

BioMag Laboratory, HUS Diagnostic Center, University of Helsinki and Helsinki University Hospital, Helsinki, Finland.

**Methods:**

High-PAS was applied for 4 weeks to 5 patients with incomplete, non-traumatic SCI and chronic neuropathic pain in upper limb(s). Median, ulnar, and radial nerves of the more painful hand were stimulated. The same patients also received sham stimulation for 4 weeks. Pain was measured with Verbal Rating Scale weekly and with Brief Pain Inventory before and after both stimulation periods and after follow-up of 8 weeks.

**Results:**

Clinically significant relief in pain was not achieved with high-PAS or sham treatment.

**Conclusions:**

In this pilot study, clinically significant pain relief was not observed with high-PAS compared with sham treatment. Larger studies are needed to confirm these findings. Nevertheless, pain is not a contraindication for high-PAS in rehabilitation. The previously reported positive effect on milder neuropathic pain may be due to improved muscle activity, different pain types, or placebo effect. High-PAS targeting sensory tracts instead of motor tracts merits further investigation for pain treatment.

**Trial registration:**

clinicaltrials.gov, ID NCT05362422

## Introduction

Spinal cord injury (SCI) is a complex condition with significant physiological, psychological, and social effects on an individual’s life [1]. SCI symptoms depend on the extent and severity of the lesion. They can include incomplete or complete loss of sensory or motor control and autonomic regulation of the body, often leading to disability [1]. Chronic pain is a common complication after SCI, with an estimated prevalence of 53% of all patients with SCI [2]. Post-SCI pain is often severe and considerably affects quality of life [3]. Patients with post-SCI pain experience stress and depressive symptoms more often than patients without pain [4]. Neuropathic pain is the most typical pain type after SCI [3]. The pain is classified as neuropathic if it includes sensory deficits or abnormal responses to either normally non-painful stimulus (allodynia) or painful stimulus (hyperalgesia) and one or more descriptions of abnormal unpleasant or painful sensation, such as ‘hot-burning’, ‘tingling’, ‘pins and needles’, or ‘painful cold’ [5, 6]. Post-SCI neuropathic pain can occur at injury level (at-level), where the pain is located between one dermatome above and three dermatomes below the neurological level of injury, or below level, where the pain is located more than three dermatomes below the neurological level of injury [7].

The exact mechanism of the development of neuropathic pain in some SCI patients is unknown. It may follow altered sensory processing at any point along the path from peripheral sensation to conscious perception [7]. Changes in spinal cord neuronal firing with altered neurotransmitter levels and glial activation may generate pain [7]. At-level pain may relate to inflammatory and neurochemical changes of the nerve cells surrounding the site of injury, causing increased responsiveness to peripheral stimulation or neuronal hyperexcitability [8, 9]. Below-level pain may result from activation of inflamed damaged axons in the residual spinothalamic tract [10].

Currently post-SCI pain is mainly treated pharmacologically. Treatments include antidepressants, anticonvulsants, and opioids. Such treatments may have side effects and are not particularly effective [11]. Effective non-pharmacological treatment approaches for post-SCI pain are limited [11, 12]. Although transcranial direct current stimulation or exercise programs may provide reduced pain in the short- or mid-term, other methods have either failed to reduce post-SCI pain or lack high-quality studies [12, 13]. Repetitive transcranial magnetic stimulation (TMS) has been successful in the treatment of other types of chronic pain, but the results have been less promising with post-SCI neuropathic pain [14, 15]. Some patients with SCI might still benefit from a series of repetitive TMS treatment [15].

Paired associative stimulation with high-intensity TMS and high frequency peripheral component (high-PAS) is a novel therapeutic tool under investigation for patients with SCI. High-PAS improves motor deficits and also ameliorates post-SCI neuropathic pain in some patients [16, 17]. High-PAS is based on activation of a wide net of neural circuits, which is achieved by producing several synchronous antidromic and orthodromic stimuli [16, 18]. More specifically, high-PAS aims to induce plasticity at the spinal level of the corticospinal tract and activate motor nerves [16, 19]. Sensorimotor cortical activity is also modified [20]. The aim of this study was to investigate whether high-PAS stimulation can alleviate pain in SCI patients with neuropathic pain occurring in the upper limb and compare the results with sham treatment in the same patients.

## Methods

### Patients

Seven patients (4 females and 3 males, mean age 59.7 SD ± 7.1 years) from Helsinki University Hospital Spinal Cord Injury Center and the Finnish Spinal Cord Injury Register were recruited. Inclusion criteria were cervical-level SCI, age between 18–70 years, time since injury >18 months, and chronic unilateral or bilateral neuropathic pain in upper extremities. Patients who had brain injury, epilepsy, high intracranial pressure, metal implants in the head area, or implanted electrical devices were excluded. All patients were ambulatory with incomplete, non-traumatic injury (American Spinal Cord Injury Impairment Scale=D). One patient was excluded after magnetic resonance imaging revealed a small brain contusion. Another patient decided to withdraw before the first stimulation due to personal reasons. Five patients completed the entire intervention as planned. Their rehabilitation program and medications were not changed during the study.

The extent of neuropathic pain was confirmed with the Pain Detect questionnaire [21]. Patients 1–3 had clear positive results from this questionnaire (>19/38 points). Although the results were ambiguous in patients 4 and 5 (15/38 and 12/38 points, respectively), these patients were included because other criteria for neuropathic pain were met. Differentiation of at-level and below-level pain was challenging in patients 2–5 because the neurological level of injury was defined above the lesion site due to sensory deficits in American Spinal Injury Association (ASIA) sensory testing, but the pain site and most painful dermatome correlated with the lesion site. Detailed information on patient characteristics is presented in Table 1.

**Table 1:** Patient characteristics.

Patient	Age	Sex	Injury characteristics	AIS and NLI	Pain	Pain and other CNS-active medication
**1**	69	F	Intramedullary cavernoma and haemorrhage at C1-C4 level. Time since injury 3 y 7 mo.	AIS = D, NLI = C1	Combination of at-level and below-level pain in left upper limb, fingers.	Duloxetine 90 mg Buprenorphine 5 µg/h Paracetamol 1000–3000 mg on demand
**2**	61	M	Cervical spinal stenosis and medullopathy at C5-C6, operated 2016. Time since injury 6 y.	AIS = D, NLI = C3	At-level pain on right and left, ulnar side of forearm, and hand.	Buprenorphine 0.4–1.6 mg on demand Pregabalin 450 mg Tramadol 50–150 mg on demand
**3**	53	F	Cervical spinal stenosis and medullopathy at C6/7, operated 2014. Time since injury 7 y 7 mo.	AIS = D, NLI = C1	At-level pain on right and left radiating from neck to ulnar side of forearm and hand.	Gabapentin 2700 mg Duloxetine 90 mg Paracetamol/codeine 500/30 on demand Zolpidem 10 mg on demand
**4**	62	M	Cervical spinal stenosis and medullopathy at C5, operated 2013, 2019, 2021. Time since injury 10 y.	AIS = D, NLI = C1	At-level pain on left upper arm and forearm/hand	Buprenorphine 0.5 mg on demand
**5**	60	F	Cervical spinal stenosis and medullopathy at C4-C5, operated 2019. Time since injury 3 y 2 mo.	AIS = D, NLI = C1	At-level pain in left wrist and upper arm.	Pregabalin 300 mg Baclofen 10–20 mg

Medication shown as daily doses. Time since injury counted from the haemorrhage (patient 1) or the first finding of medullopathy (patients 2–5) to start of the intervention.

*AIS*, American Spinal Cord Injury Impairment Scale, *NLI*, Neurological level of injury (determined by the sensory testing of the American Spinal Injury Association examination sheet), *CNS-active*, Central nervous system active.

Patients were informed that the study included two 4-week stimulation periods, and the given stimulation was randomized to a) regular high-PAS, b) sham stimulation, or c) regular high-PAS with different settings. No stimulations with type c were delivered. This measure was implemented to ensure patients could not differentiate between sham and high-PAS. The study was approved by Helsinki University Hospital Regional Committee on Medical Research Ethics (HUS/1280/2016) and was performed in accordance with the Declaration of Helsinki. All participants provided written informed consent for participation. This study was registered at clinicaltrials.gov (NCT05362422).

Expectations for treatment were screened with questionnaires before sham- and high-PAS periods. This questionnaire assessed the anticipation of the patients on the effects on pain treatment and functional ability. Patients were asked whether they thought they had received regular or sham stimulation after each stimulation period.

### Experimental setup

**Fig. 1:**
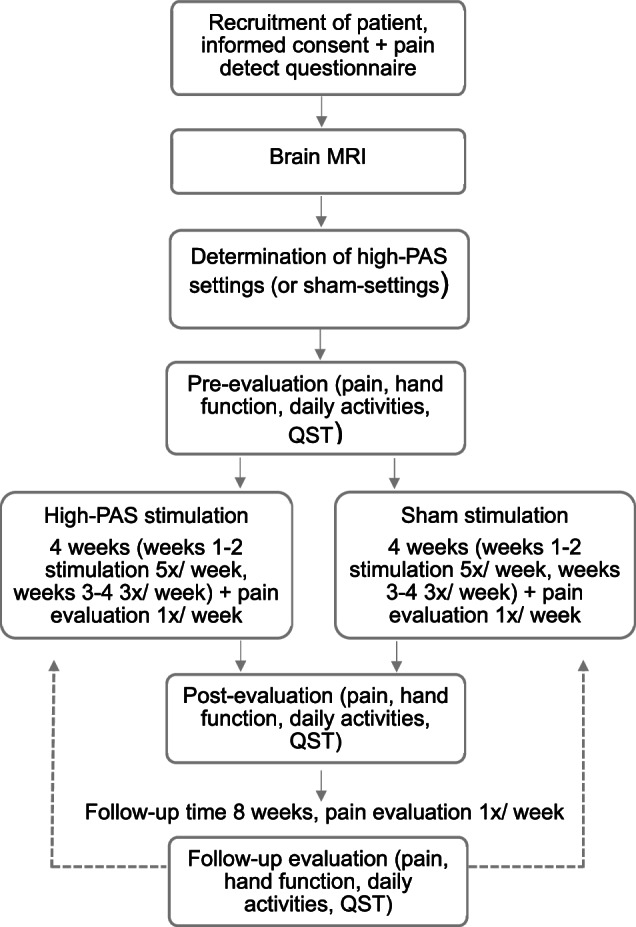
Experimental setup. After the first stimulation period (high-PAS/sham) and 8 weeks follow-up, patients started another stimulation period (sham/high-PAS). 1^st^ follow-up evaluation = 2^nd^ pre-evaluation. MRI=magnetic resonance imaging, QST=quantitative sensory testing.

The study consisted of two 4-week stimulation periods of regular high-PAS and sham stimulation that were administered in random order. The hand with more pain was stimulated. Before the first stimulation period, patients underwent magnetic resonance imaging for TMS-navigation and safety reasons. The experimental setup is illustrated in Fig. 1.

### Stimulation settings

TMS was administered with an eXimia magnetic stimulator with a navigated figure-of-8 coil (Nexstim Ltd., Helsinki, Finland). Cortical representations of the abductor digiti minimi muscle representing ulnar nerve, the abductor pollicis brevis muscle representing median nerve, and the extensor digitorum communis muscle representing radial nerve were mapped. The TMS ‘hotspot’ for each muscle was determined individually as the site where TMS elicited the largest motor-evoked potentials (MEP). MEP latency for interstimulus interval calculation was measured from an average of 15 MEPs elicited by TMS of 100% stimulator output. Peripheral nerve stimulation (PNS) was delivered, and F-responses were measured with a Keypoint device (Natus Medical Inc., Pleasanton, CA, USA). F-responses for ISI calculation were measured at supramaximal stimulation intensity with a 0.2-ms pulse for determining minimum latency out of 10 responses [22]. Thereafter, minimum current evoking F-responses was determined with a 1-ms pulse, and this pulse duration and current intensity were used for high-PAS. Details on stimulation parameters are presented in Table 2.

**Table 2:** Stimulation parameters.

Patient	Stimulated hand	Stimulation period order	MEP latency (ms)	F-latency (ms)	PNS intensity (mA)	ISI (ms)
**1**	left	sham first				
median			21.9	26.5	2.0	4.6
ulnar			22.3	26.3	7.0	4.0
radial			18.6	18.0	8.0	−0.6
**2**	left	sham first				
median			24.7	35.0	6.0	10.3
ulnar			28.3	33.5	10.0	5.2
radial			16.9	21.0	4.5	4.1
**3**	right	high-PAS first				
median			23.4	30.5	3.5	7.1
ulnar			23.9	27.0	3.0	3.1
radial			14.3	21.0	22.0	6.7
**4**	left	sham first				
median			27.0	36.8	2.5	9.8
ulnar			27.5	35.1	3.0	7.6
radial			17.0	19.8	6.5	2.8
**5**	left	high-PAS first				
median			30.8	30.8	3.5	0.0
ulnar			24.1	29.5	3.0	5.4
radial			13.7	21.3	9.0	7.6

*MEP*, motor evoked potential, *PNS*, peripheral nerve stimulation, *ISI*, interstimulus interval.

Self-adhering surface electrodes (Neuroline 720, AMBU A/S, Ballerup, Denmark) were used for PNS, F-response measurements, and MEP recordings. During stimulation, electrodes were gently pressed to improve nerve activation. The same electrode setting was used for F-response measurement and PNS. Two recording electrodes were placed at a belly-tendon montage over abductor pollicis brevis, abductor digiti minimi, and extensor digitorum communis representing median, ulnar, and radial nerves, respectively, in F-response measurements and MEP recordings (Supplementary file).

High-PAS consisted of a single-pulse TMS at 100% of stimulator output and PNS in 100-Hz trains of 6 pulses (pulse width 1 ms) that were paired every 5 s (0.2 Hz). In previous studies, the 100-Hz PNS pulse train was more effective than higher or lower frequencies [23, 24], and a 0.2-Hz frequency for paired pulses produced better outcome than 0.4 Hz [24]. Interstimulus interval was calculated individually for each subject with the formula *Interstimulus interval* = *F-response latency - MEP latency* to provide synchronous arrivals of the first descending activity induced by TMS and first ascending activity induced by PNS at the spinal-cord level [25, 26]. TMS and PNS were triggered with Presentation® software (Neurobehavioral System Inc., Albany, NY, USA). One high-PAS session consisted of 240 paired pulses (20 min) per nerve (in total 60 min + preparations).

Sham stimulation was performed with the same equipment as the high-PAS. A 7.5-cm thick plastic tube was used below the coil to diminish the induced electric field close to zero in the brain. In sham PNS, the electrodes were placed away from the nerves based on their anatomical course as follows: in the middle of the dorsal side of the wrist for median nerve, in the middle of the forearm on the radial side for ulnar nerve, and in the distal third of the back side of the upper arm for radial nerve. This approach is comparable to a sham-controlled neuromodulation study using tibial nerve stimulation [27]. PNS was performed through electrodes at sham positions (trains of three 40-µs pulses at 3 Hz) at intensity just about sensory threshold, which was individually determined at pre-stimulation measurements, to ensure that stimulation caused subjective sensation but did not reach motor nerves [28]. A sham session had the same duration and number of TMS-pulses as a regular high-PAS session. Light voluntary target muscle activation (little finger abduction for abductor digiti minimi, abduction and flexion at the carpometacarpal joint of the thumb for abductor pollicis brevis, and wrist and finger extension for extensor digitorum communis) was included in both sham and high-PAS stimulation.

### Outcome measures

The primary outcome was subjective pain measured with Verbal Rating Scale (VRS) [29] weekly during the intervention and with the Brief Pain Inventory (BPI) [30] before and after both stimulation and follow-up periods. Patients reported by text message or phone call a number between 0 and 10 (10 indicating the most severe pain) that described their average pain intensity in the stimulated hand during the previous week. Additionally, VRS data were collected before and after every stimulation session. Pain relief is considered clinically significant if pain decreases by 30% [31]. Primary outcome measures were complemented with the International SCI Pain Basic Data Set (21), Pain Anxiety Symptom Scale Short Form 20 [32], and items considering Energy and feelings and Quality of life and general health from International Spinal Cord Injury Survey. These secondary pain-related outcome measures were not pre-registered at clinicaltrials.com [3].

Functional abilities of the patients were evaluated using the Spinal Cord Injury Independence Measure [33] and the Disabilities of the Arm, Shoulder and Hand [34] self-reporting questionnaires. Hand function was measured using the 9-Hole Peg test [35] and Box and Block test [36]. Modified Ashworth Scale was used to evaluate spasticity [37]. Hand and arm muscle strength was tested with Medical Research Council scale (0–5) manual muscle testing [38]. Additionally, strength of the key hand muscles (C2-C8, T1) and sensory scores (C2-C8, T1-T2) were tested using the ASIA examination sheet [39]. Light touch and pin-prick sensory tests were performed. Grip strength was measured with an Exacta™ Hydraulic Hand Dynamometer (North Coast Medline, Inc., USA) and pinch dynamometry with a Baseline® Mechanical Pinch Gauge (Fabrication Enterprises Inc., USA) for key pinch, tip pinch, and palmar pinch [40]. The hand-function tests and physical evaluation were performed by the same experienced physiotherapist for all patients. The physiotherapist was not aware of the type of the stimulations. After every stimulation period, the physiotherapist was asked whether they thought the patient received high-PAS or sham stimulation.

A quantitative sensory test (QST) was performed on patients to measure cold detection threshold, warm detection threshold, cold pain threshold, heat pain threshold, and vibration detection [41]. Testing spots were located in the area of pain and in the dermatome innervated by stimulated nerve as follows: C8 dermatome, proximal phalanx of V finger in patients 1 and 2; C7 dermatome, proximal phalanx of III finger in patients 3 and 4; and C6 dermatome, proximal phalanx of I finger in patient 5. QST was performed by an experienced nurse in Clinical Neurophysiology Outpatient Clinic in Helsinki University Hospital. The nurses were not aware of the type of stimulation (high-PAS or sham).

Verbal feedback was collected throughout the study. The patients were asked to describe the nature of pain and any possible changes. Adverse events were also collected.

### Statistical analysis

All group-level results are presented as mean ± standard error. In the original trial registration, both descriptive and inferential analyses were planned to assess differences between stimulation conditions. However, due to recruitment challenges, only 5 patients completed the study. Given this small sample size and the high individual variability, we limited the analysis to descriptive statistics and visual inspection of individual changes, which did not show consistent trends or notable differences between high-PAS and sham stimulation. Therefore, no formal statistical testing was performed.

## Results

### Pain

**Fig. 2:**
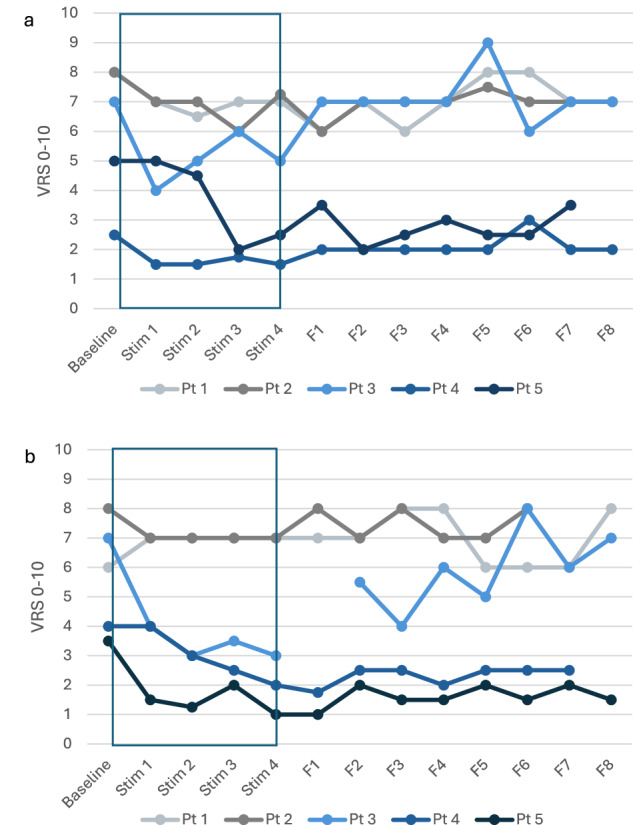
a) Verbal Rating Scale (VRS) fluctuation during high-PAS period per patient; b) Verbal Rating Scale (VRS) fluctuation during sham period per patient. Stim = stimulation week, F = follow-up week, Pt = patient, blue box = stimulation period.

Weekly VRS values fluctuated slightly during the treatment periods (Fig. 2a, b). Fluctuations were less than three units from baseline before starting the intervention. Overall, the reported pain level was reduced by the end of the high-PAS period in all patients by a mean of 1.45 ± 0.3 units (28%) compared with baseline. No pain increases occurred. After the follow-up period, mean reduction in the reported pain level was 0.8 ± 0.3 units (15%). By the end of the sham period, the reported pain level was reduced by a mean of 1.7 ± 0.8 units (35%) compared with baseline. After the sham follow-up period, mean decrease in reported pain level was 0.3 ± 0.7 units (9%).

The mean decrease in BPI pain total score of the stimulated hand was 13.4 ± 7.4 units (22%) after high-PAS. After the follow up, mean decrease in the pain index was 12.8 ± 10.8 units (19%). After the sham period and follow up, the mean BPI total score decreased by 10.2 ± 5 units (6.5%) and 10.8 ± 4.8 units (11%), respectively. The pain intensity and pain interference domains of BPI acted slightly differently between high-PAS and sham periods, but individual variability between the patients was notable. The mean intensity score was 5.45 ± 1 before high-PAS, 4.85 ± 1 after high-PAS, and 4.85 ± 1 after follow-up (decrease 11% from the baseline). With sham treatment, the corresponding scores were 5.09 ± 0.9, 3.95 ± 0.7 (decrease 22%), and 4.3 ± 1 (decrease 15%). In the pain interference domain, which measures how much the pain interferes with daily activities, the mean score decreased from 4.48 ± 0.9 to 3.14 ± 1 (30%) after high-PAS and from 4.29 ± 1 to 3.34 ± 0.5 (22%) after sham. The decrease was 28% from the baseline after follow up of both periods.

Pain Anxiety Symptom Scale results were consistent with BPI; pain-related anxiety increased in the patients whose pain did not decrease after the high-PAS or sham period. Mean Pain Anxiety Symptom Scale score decreased 1.4 ± 4.2 points after high-PAS and 11.6 ± 7.1 points after sham period. In the International SCI Pain Basic Data Set, the mean overall score was 21.8 ± 2.6 at baseline, 20.6 ± 1.6 after high-PAS, and 19.4 ± 2.4 after follow up. The corresponding values after sham treatment were 19.6 ± 2.4, 16.8 ± 1.1, and 19.6 ± 2.1, respectively.

### Secondary outcomes

At baseline, 4/5 patients had normal power (5 on Medical Research Council scale) in all muscles tested with manual muscle testing. One patient had decreased strength in the stimulated hand (mean of tested muscles 2.8 on Medical Research Council scale) at baseline and the strength remained unchanged throughout the intervention. In the 9-Hole Peg test of finger dexterity, 4/5 patients had maximum value (18/18) already at baseline. No change in other physical or functional tests was observed. Quality of life remained unchanged (Table 3).

**Table 3:** Secondary outcomes.

Test	Baseline high-PAS	Post high-PAS	Follow-up high-PAS	Baseline sham	Post sham	Follow-up sham
MRC (points)^a^	4.54 ± 0.5	4.57 ± 0.4	4.58 ± 0.4	4.56 ± 0.4	4.5 ± 0.5	4.55 ± 0.5
Grip strength (kg)	27.2 ± 6.7	24.6 ± 7.1	27.3 ± 6.8	26.2 ± 6.7	27.7 ± 6.9	27.2 ± 6.7
Tip pinch (kg)	4.24 ± 1.2	3.7 ± 1.2	4.59 ± 1.3	4.34 ± 0.7	4.3 ± 0.6	4.09 ± 1.2
Key pinch (kg)	6.45 ± 1.2	6.05 ± 1.4	6.7 ± 1.2	7 ± 0.8	6.7 ± 1	6.05 ± 1.3
Palmar pinch (kg)	5.39 ± 1.4	4.9 ± 1.2	5.4 ± 1.3	5.3 ± 0.8	5.35 ± 1	5.09 ± 1.5
BBT (pcs)	54.4 ± 6.9	54 ± 7.3	53 ± 8.1	52 ± 6.8	53.8 ± 6	54.8 ± 6.7
9-HPT (pcs)^a^	14.4 ± 3.6	14.6 ± 3.4	14.8 ± 3.2	14.8 ± 3.2	15.2 ± 2.8	14.4 ± 3.6
MAS (points)^a^	1,9 ± 1.9	1,3 ± 1.3	1,2 ± 1.2	1,6 ± 1.4	1,7 ± 1.7	1,9 ± 1.9
ASIA UEMS (points)	48.2 ± 1.8	48.2 ± 1.8	48 ± 2	48.4 ± 1.6	47.8 ± 2.2	48.2 ± 1.8
ASIA LT (points)	30.4 ± 2.6	29.8 ± 2.5	27.8 ± 2.4	26.6 ± 1.7	28 ± 2.8	32 ± 2.1
ASIA PP (points)	24.6 ± 2.8	23.8 ± 2.6	24 ± 3.4	23.2 ± 2.6	23.2 ± 2.3	25.4 ± 3.1
DASH (points)	53,7 ± 9.7	49,3 ± 9.6	46,9 ± 10	50,63 ± 10	51,5 ± 10.5	50,53 ± 8.7
SCIM (points)	90,6 ± 5.3	90,8 ± 5.2	90,4 ± 5.4	90,8 ± 5.2	90,4 ± 5.4	90,6 ± 5.3
Energy & feelings (points)	26.4 ± 1.3	27 ± 0.9	26.2 ± 0.8	25.2 ± 0.6	26.6 ± 1.4	26 ± 1.7
Quality of life (points)	24.2 ± 0.9	24 ± 1.7	25.2 ± 1	24.2 ± 0.7	24.8 ± 1.3	24 ± 0.8

All results presented as mean ± standard error.

*MRC*, Medical Research Council Scale Manual muscle test, *BBT*, Box and Block test, *9-HPT*, 9-Hole Peg test, *MAS*, Manual Ashworth Scale, *ASIA*, American Spinal Injury Association, *UEMS*, Upper extremity motor score, *LT*, Light touch, *PP*, Pin prick, *DASH*, Disabilities of the Arm, Shoulder and Hand, *SCIM*, Spinal Cord Injury Independence Measure.

^a^All patients except pt 1 had normal score in baseline evaluations. Stimulated hand was tested.

No significant group-level changes, differences between stimulation periods, or differences between the stimulated and non-stimulated hand were observed in QST variables. Individual-level changes were detected in cold pain threshold perception. Before high-PAS, 4 patients had lower and one patient significantly higher cold pain threshold than healthy individuals of the same gender and age [42]. In 3 patients with lower threshold, the cold pain threshold increased towards normal after high-PAS by 9.4 ± 4.8˚C but decreased in the other 2 patients by 4.2 ± 1.0˚C. Overall, cold pain threshold changed towards normal after high-PAS in 4/5 patients. A similar trend was detected after sham stimulation in the same hand (+11.8 ± 4.6˚C in 4/5 patients) but not in the contralateral hand (1.3 ± 5.8˚C). Changes in other variables were seen only in patient 1.

### Adverse events and verbal feedback

Patients experienced a maximum pain relief of 2 VRS units from before to after high-PAS. All patients reported that they were “feeling better” after high-PAS on at least 2 sessions out of 16. Pain increases during stimulation were rare. Differences were not notable between high-PAS and sham stimulation. Patient 5 experienced a decrease in “tickling” sensation related to her pain after the first week of high-PAS. Patient 3 reported less pain in her hand in the evenings and weekends during both high-PAS and sham stimulation than before treatments or during follow-up. Pain in patient 2 increased during the first sham stimulation sessions but this was eliminated by omitting the pre-activating movements. Motor imagery was used instead. Other adverse events were not reported. Although some patients considered the high-PAS uncomfortable at first, they got used to induced sensations after a few sessions.

### Expectations to treatment

Before high-PAS, the mean expectation to treatment index was 9.6/20, indicating that the patients most frequently responded “partially agree” to the statements given. Before the sham period, the mean expectation index was 11.4/20, indicating slightly higher expectations. Three of five patients recognized high-PAS as actual stimulation but none of the patients recognized sham; 2 patients believed they had actual stimulation, and 3 patients could not tell. The physiotherapist recognized high-PAS in 3 patients and sham in one patient.

## Discussion

This was the first sham-controlled study investigating the effect of motor-tract high-PAS on non-traumatic post-SCI neuropathic pain. Overall, no differences between high-PAS and sham on perceived pain were observed. Pain relief is considered clinically significant if the pain decreases by 30% [28]; this was not achieved at the group level. Considering the small sample size, larger sham-controlled studies with more patients are needed to confirm this result. However, this result is consistent with previous studies conducted with repetitive TMS [14] and spinal-cord stimulation [13]; neuromodulation treatments are less effective in treating severe post-SCI pain than other forms of chronic pain. On the other hand, high-PAS did not increase the pain, and even patients with the most severe pain tolerated the stimulation. Thus, severe neuropathic pain should not be a contraindication to high-PAS if the patient is otherwise expected to benefit from high-PAS in motor recovery. In previous high-PAS studies aimed at motor rehabilitation, altogether 6 out of 7 patients with mild-to-moderate neuropathic pain reported decreased pain [16] and a case report described a positive effect of high-PAS in severe traumatic post-SCI neuropathic pain [17]. Although the placebo effect may have contributed to pain relief in previous studies, other explanations are also plausible.

In previous studies, reduced pain was associated with improved motor function and hand muscle strength. On the other hand, increasing pain may lead to motor deficits. Although neuropathic pain relief along with improvements in motor function has been reported in patients with SCI following exercise programs [43, 44], the relationship between these two outcomes remains unexplored. Pain itself produces complementary, additive, or competitive changes at multiple levels of the motor system and activity is re-organized within and between muscles [45]. It is possible that decreased pain after high-PAS is linked to improved muscle activity related to high-PAS induced plasticity at corticomotoneuronal synapses of the spinal cord and normalized pathway signalling. As 4/5 patients in this study had normal motor activity at baseline, it was not possible to induce further improvement. It is not clear why motor activity did not increase in one patient who had deficiencies in muscle strength and hand function. It is notable that patients who have previously benefited from high-PAS in motor function did not have severe neuropathic pain [46–48], except for one patient documented as a case report who had a different pain type that also included nerve root damage [17]. Future studies should include a larger cohort of patients with both post-SCI pain and motor deficits and longitudinally assess their pain levels to better elucidate the relationship between motor recovery and pain alleviation.

All patients received central nervous system active medications (opioids, gabapentinoids, or duloxetine) to treat pain. These patients used stronger pain medications, especially opioids (such as buprenorphine), at greater doses than patients in previous high-PAS studies. Both gabapentin and pregabalin might inhibit the effects of PAS through increased gamma-aminobutyric acid synthesis in the motor system [49]. In a previous case study, pregabalin did not block the positive effect of high-PAS in pain or in motor function [17]. Opioids or duloxetine are not known to affect PAS-induced plasticity. Nevertheless, the possible negative effects of these medications and their combinations cannot be excluded. The analgesic effects of repetitive TMS on chronic pain are thought to be mediated by the activation of endogenous opioid systems in the brain [50]. Therefore, it is advisable to reduce or temporarily discontinue opioid medications during the repetitive TMS treatment period to minimize potential interactions [51]. In this study, patients continued their opioid medications without changes, as discontinuation of pain medication would have been both unethical and could have created withdrawal symptoms, which could have further affected the results. It is also important to determine how treatments such as high-PAS work in real-life situations.

QST values were not notably modified by high-PAS. This is the first time that QST results were reported in this context. In previous studies, sensory function was measured by ASIA sensory scores (light touch/pin prick) and no changes were observed [16]. Thus, the current results with a more sophisticated tool are consistent with previous results. Consequently, motor-tract high-PAS appears to be specific to motor pathways. Stimulation of primary or secondary sensory cortical areas instead of primary motor cortex might provide different results for pain and sensory function, and this topic warrants further investigation.

The limitations of this study were the low number of patients, the lack of statistical analysis, the duration of the stimulation period, and the short wash-out time between the two stimulation periods. In previous studies, motor function and modulation of sensorimotor oscillations improved [20, 46] and pain was reduced [17] within 4 weeks, although some patients may require longer high-PAS periods for full benefit [47]. In patients 3 and 5, high-PAS preceded sham stimulation, and the first period could have affected the results of the second one due to the short wash-out time.

To conclude, clinically significant pain relief was not observed with high-PAS compared with sham treatment. Larger, well-powered studies are necessary to validate these findings. Nonetheless, neuropathic pain should not be considered a contraindication for the use of high-PAS in rehabilitation settings. Future research should explore high-PAS protocols that specifically target sensory pathways rather than motor tracts for potential applications in pain management.

## Supplementary information

Supplementary file

## Data Availability

The datasets generated and/or analysed during the current study are available from the corresponding author on reasonable request.
