# Supplementary material for: Paired associative stimulation with a high-intensity cortical component and a high-frequency peripheral component in treatment of neuropathic pain after incomplete spinal cord injury – a pilot trial
Source: Spinal Cord Ser Cases. 2026 Mar 4;12:3. doi: 10.1038/s41394-026-00729-1 (PMC12957355; doi:10.1038/s41394-026-00729-1)
Supplement: Supplementary file 1 — Supplementary file [file 41394_2026_729_MOESM1_ESM.pdf]

Supplementary file

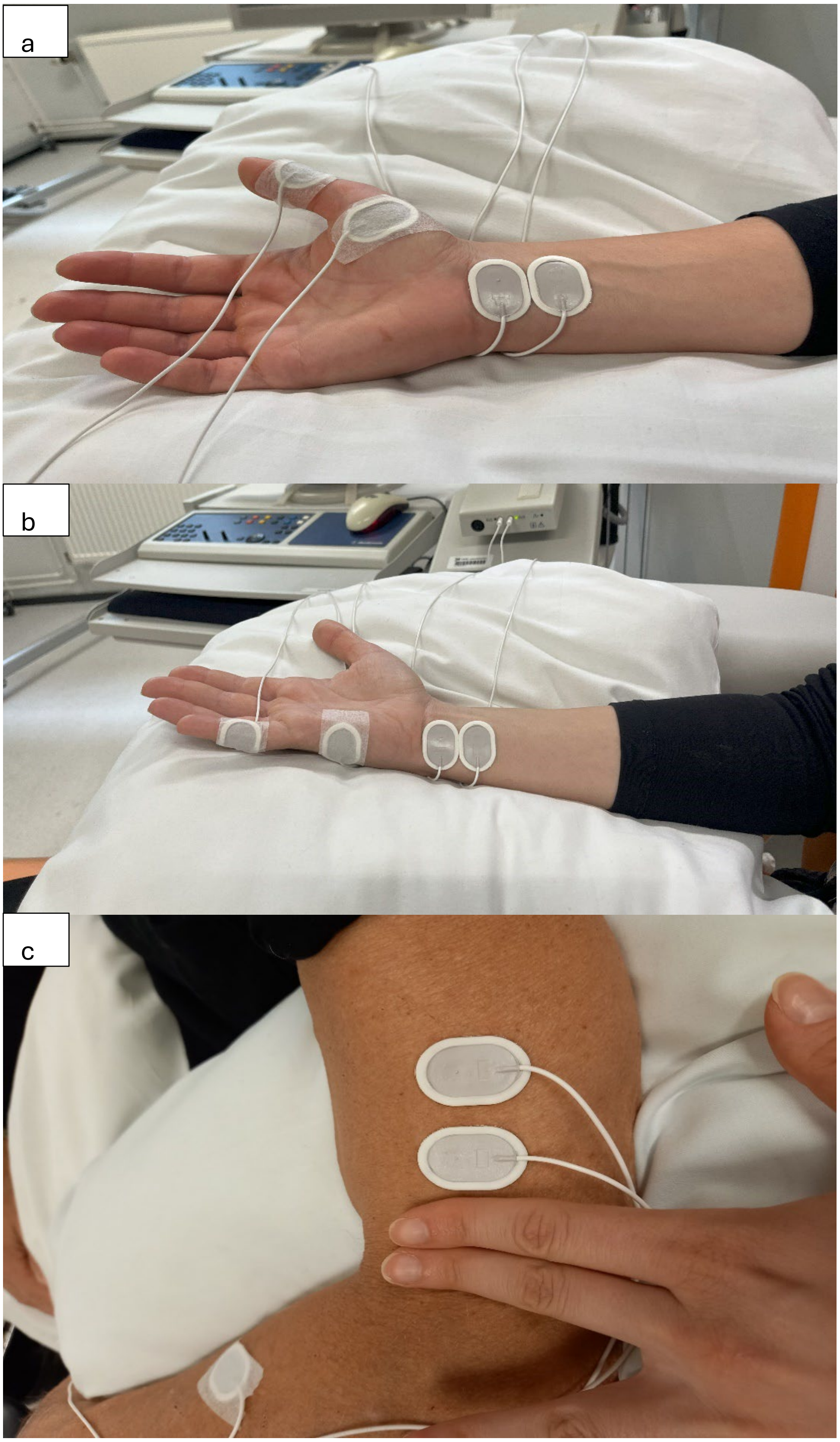

a) Electrode placement for median nerve active stimulation and F-response measurement;
b) Electrode placement for ulnar nerve active stimulation and F-response measurement;
c) Electrode placement for radial nerve active stimulation and F-response measurement.
